# Autopsy of Used Reverse Osmosis Membranes from the Largest Seawater Desalination Plant in Oman

**DOI:** 10.3390/membranes12070671

**Published:** 2022-06-28

**Authors:** Mohammed Al-Abri, Htet Htet Kyaw, Buthayna Al-Ghafri, Myo Tay Zar Myint, Sergey Dobretsov

**Affiliations:** 1 Nanotechnology Research Center, Sultan Qaboos University, P.O. Box 33, Al-Khoudh, Muscat 123, Oman; htet@squ.edu.om (H.H.K.); b.alghafri2@squ.edu.om (B.A.-G.); 2 Department of Petroleum and Chemical Engineering, College of Engineering, Sultan Qaboos University, P.O. Box 33, Al-Khoudh, Muscat 123, Oman; 3 Department of Physics, College of Science, Sultan Qaboos University, P.O. Box 36, Al-Khoudh, Muscat 123, Oman; myomyint@squ.edu.om; 4 Department of Marine Science and Fisheries, College of Agricultural & Marine Sciences, Sultan Qaboos University, P.O. Box 34, Al-Khoudh, Muscat 123, Oman; sergey@squ.edu.om; 5 Center of Excellence in Marine Biotechnology, Sultan Qaboos University, P.O. Box 50, Al-Khoudh, Muscat 123, Oman

**Keywords:** seawater reverse osmosis membrane, desalination plant, fouling, scaling, bacteria, autopsy

## Abstract

The Barka desalination plant, commissioned in 2018, is the largest desalination plant in Oman. It has a capacity of 281 MLD with a reverse osmosis (RO) first-pass recovery rate of 46%. As part of the standard operator practice, a membrane autopsy was conducted to determine the cause of reductions in membrane performance. This study investigated fouled membranes (model No. SW30HRLE-440) from two different locations in the membrane rack. Various analytical methods were used to conduct the membrane autopsy. Field-emission scanning electron microscopy/energy-dispersive X-ray (FESEM/EDS) analyses of membrane samples showed major components of inorganic foulants. Moreover, black and salt-like crystals deposited on the membrane surface revealed significant carbon (C) components and oxygen (O), with a small amount of magnesium (Mg), chloride (Cl), sodium (Na), aluminium (Al), and calcium (Ca), respectively. A Fourier transform infrared (FTIR) analysis revealed the presence of long-chain hydrocarbons, carboxylic acids/esters, carbohydrates/polysaccharides, and inorganic foulants. Thermogravimetric analyses (TGA) of the membranes showed a high initial weight loss due to organic and inorganic fouling. X-ray photoelectron (XPS) analyses further confirmed the presence of inorganic and organic foulants on the membrane surfaces. Bacteria identification results showed the presence of Bacillus cereus and Bacillus marisflavi. This paper offers a detailed analysis of the foulants present on the reverse osmosis membrane surface and sub-surface before and after a cleaning process.

## 1. Introduction

Reverse osmosis desalination is widely used for seawater desalination and for treating advanced wastewater. A standard SWRO technology is the most energy-efficient option for desalination, with a total cost of energy consumption of 1.5–5.5 kWh/m^3^; this is significantly lower than other technologies [1,2,3]. The SWRO membrane lifetime depends on raw water quality, hydraulic operation conditions, membrane fouling, and cleaning frequency. Membrane fouling refers to the deposition of unwanted materials on or inside a membrane’s surface or its pores. Due to their nonporous structure, surface fouling occurs in RO systems more than in other membrane filtration processes [4,5]. 

Foulants are categorized into four major groups: soluble inorganic materials, colloidal matter, dissolved organic contaminants, and biological matter. It is important to note that while the distinction between inorganic, colloidal, organic, and biological foulants is useful, RO membranes are likely to encounter all four categories of foulants during a typical RO operation. Soluble inorganic compounds and scaling cause inorganic fouling. It occurs when dissolved salts in the feed water precipitate on the membrane due to an increased salt concentration that exceeds the solubility limit. García-Triñanes et al. [6] identified several types of inorganic scaling, such as silica (SiO_2_), sulphate (SO_4_^2−^), carbonate (HCO_3_^−^), calcium (Ca), iron (Fe), sodium chloride (NaCl), and aluminium (Al). Al may be present due to Al_2_(SO_4_)_3_ coagulant used in the SWRO system.

Upon filtration, the dispersed colloidal matter may precipitate out of the film, forming a cake layer, which creates a barrier that hinders filtration and causes colloidal fouling [7]. This exacerbates the issue of high treatment costs and reduces the quality and quantity of treated water. The complexity of colloidal fouling makes it challenging to analyze using a simple fouling index. Accordingly, Ju and Hong [8] investigated the effect of colloidal fouling in SWRO membrane flux using both a theoretical approach such as cake resistance simulator-modified fouling index nanofiltration (MFI-NF_CRS_) and experimental approaches using a dead-end NF process. 

Organic fouling results from interactions between the membrane surface and the organic foulants and between the organic foulants themselves [9,10,11]. According to Huber et al. and Jeong et al. [12,13], humic substances (HSs), low-molecular-weight neutrals (LMW-Ns), biopolymers (BPs), and building blocks (BBs) are the major foulants that affect the performance of SWRO membranes. Biofouling is the attachment and growth of microbial organisms on a membrane surface [5,14,15]. Biofouling is a more complex process compared to other fouling types. It consists of two main elements: the bacteria and extracellular polymeric substances (EPSs) that are released during bacterial metabolism [16,17]. García-Triñanes et al. [6] found that *Pseudomonas* spp. and diatoms were primarily responsible for biofouling, releasing polysaccharides containing amide (C=O), amine (N-H), and C-O groups as identified by FTIR analysis.

As a result of membrane fouling, many desalination plants face financial impacts from their mitigation and maintenance costs [1]. Despite various approaches and methods to mitigate fouling, it remains a significant operational challenge. Compared to internal fouling, surface fouling can be controlled more easily, primarily by using pre-treatment strategies. The conventional pre-treatment strategies are cartridge filtration, antiscalant (phosphonate-based antiscalant), and coagulants/flocculants addition. Hypochlorite, a strong oxidant, is used for biofouling control in the intake pipeline and is one of the most widely used disinfection substances. However, chlorine reduction is necessary (via sodium bisulfite) before it reaches the membrane to avoid chemical oxidation that damages the polyamide membrane. Some treatments increase the solubility of coagulants, and calcium carbonate causes aggregation of some organic and inorganic fouling, which can be removed later [18].

Surface fouling is more reversible than internal fouling. Nevertheless, both types of fouling can be irreversible, depending on the composition of the feed water and the interactions with the membrane [19]. Membrane fouling may result in pore blocking; the adsorption of hydrophobic particles; and the formation of nonpolar solutes, gels, or cake layers. These obstacles lead to declines in rejection and net water flux. The consequence of these problems is the short lifetime of the membrane and the need for its eventual replacement [15,20]. In contrast to low-pressure membrane fouling by surface water and groundwater, research on SWRO membrane fouling has not significantly progressed to date, despite various efforts. 

A membrane autopsy is the most effective method to understand membrane fouling. Numerous reports of RO membrane autopsies have been published, but most focused on a lab-scale or pilot-scale RO process [21,22,23]. The traditional way of assessing membrane fouling is by looking at the flux decline rate over time, but this is inadequate for evaluating deposit growth in an RO process [24,25]. For example, Tay et al. [26] noticed a significant reduction in the permeate flux of an RO membrane, indicating severe fouling occurrence. 

Membrane fouling has been investigated by analyzing autopsies to understand the underlying physico-chemical processes. Only a few limited studies have evaluated the physico-chemical processes involved in fouling [27,28,29]. The complexity of these deposits limits the value of defining fouling mechanisms from their composition. Observations using optical microscopy, including field-emission scanning electron microscopy/energy-dispersive X-ray spectroscopy (FESEM/EDXS) and X-ray diffraction (XRD), can provide insights into the surface deposits but not into the deeper deposits. This results in a lack of insight into the kinetics of deposition of various foulants and hence into the fouling mechanisms. This is especially true for thicker deposits.

Due to the complexity of fouling, several mechanistic studies simplified the problem by focusing on only one kind of foulant. Nevertheless, it is crucial to know how these different foulants interact with each other and how they affect fouling mechanisms. According to recent studies, low concentrations of salt ions in the colloidal cake layer may cause osmotic pressure to increase and also cause rapid flux to decrease during cake layer formation [27,30]. In addition, there may be interactions between foulants such as organics and colloidal particles. Some foulants act synergistically, leading to significant flux decline instead of the additive effects of organic and colloidal fouling alone [31]. 

**Figure 1:**
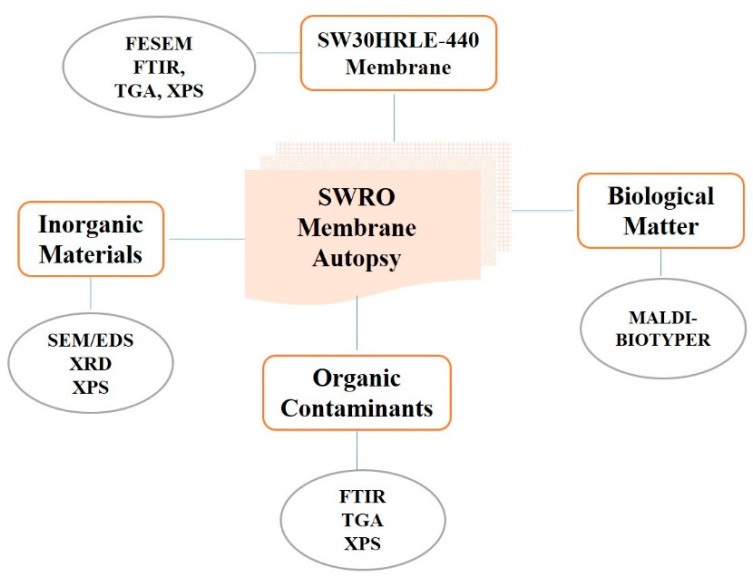
Conceptual visualization of SWRO membrane autopsy.

Membrane autopsy is useful in providing operational solutions. Additionally, protocols for the most appropriate cleaning procedure can usually be recommended once a comprehensive study of membrane fouling has been conducted during the autopsy process. The literature contains limited resources on understanding the fouling susceptibility of SW30HRLE-440 SWRO membranes [32,33]. To the best of our knowledge, an autopsy of an SWRO membrane from the Barka desalination plant has not been reported yet. This paper presents the autopsy results from studying a spiral RO membrane after nearly three years of service in a seawater desalination facility at Barka IWP, Oman. Further insights into the fouling layer’s development were gained by analyzing the top surface of the fouled membrane. The analysis of the surface deposits was carried out using morphological, chemical, and surface analysis techniques including FESEM/EDXS, Fourier transform infrared spectroscopy (FTIR), XRD, thermogravimetric analysis (TGA), and X-ray photoelectron spectroscopy (XPS). Moreover, bacteria isolation and the identification of fouled and cleaned membranes were also conducted and reported. Figure 1 shows a conceptual visualization of the content of the SW30HRLE-440 RO membrane autopsy.

## 2. Materials and Methodology

### 2.1. Plant Description and Location

**Figure 2:**
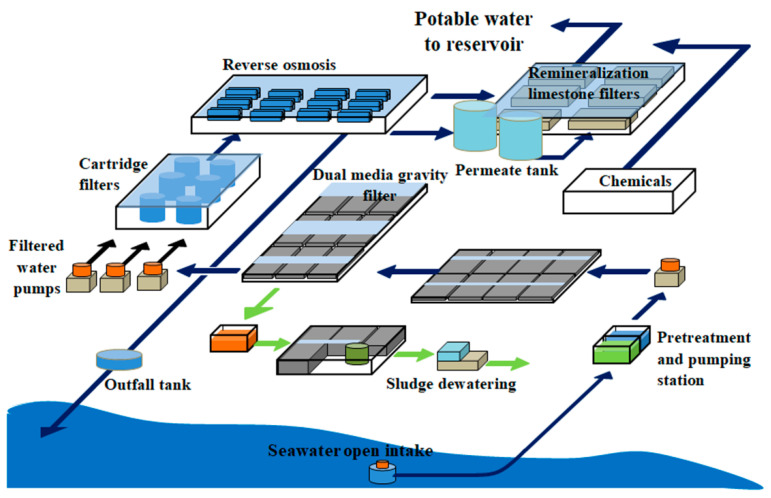
Schematic diagram of Barka IV desalination plant.

The Barka desalination plant is located 50 km west of Muscat, Oman, adjacent to the existing Batinah Coastal Highway. A schematic diagram of the desalination plant is shown in Figure 2. The plant capacity is 281 MLD with a recovery of 46% and power consumption of less than 3 kWh/m^3^.

The seawater and product water details are provided in Table 1. The plant utilizes pre-treatment techniques such as coarse and fine screening systems, dissolved air flotation (DAF) units, and dual-media gravity filters to avoid damage to RO membranes. Moreover, pre-treatment chemicals are used to ensure contractual outlet parameter values. These chemicals include antiscalant, coagulant aid (FeCl_3_), sodium hypochlorite (NaOCl), polymers, and some acids.

### 2.2. RO Membrane Autopsy

**Figure 3:**
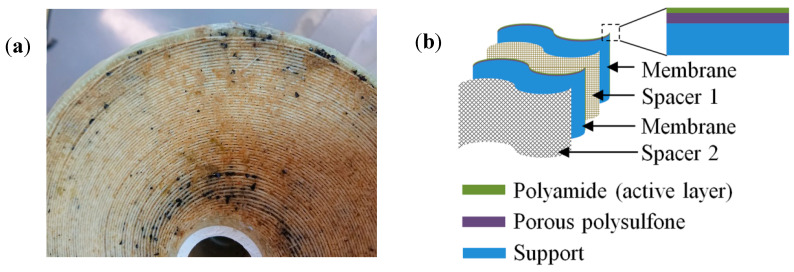
(**a**) Optical image of the cross-section of fouled spiral RO membrane cartridge. (**b**) A schematic representation of RO membrane arrangement and structure used in this work.

Three membrane modules (one new and two used) from Barka IWP, Oman, were received. The membrane supplier was Du Pont (USA) with a model No. of SW30HRLE-440. The bin/location was M1-A1-1 (1st position in the pressure vessel) for new and Rack J P11-1 and J P 11-2 (1st and 2nd position in the pressure vessel) membrane elements. The fouled SWRO membrane element selected for the autopsy study was used for nearly three years. In general, RO membrane comprises three layers [34]: (1) active layer made up of polyamide (thickness of ~10–200 nm), (2) porous polysulfone layer (thickness of ~50 µm), and (3) support layer (thickness of ~100–150 µm); this is to reinforce the membrane mechanical durability. In the spiral RO membrane cartridge, two layers of membrane are sandwiched by one layer of fine spacer (called as one pair) and each pair of membranes is separated by a thicker spacer. The basic structure of the RO membrane and multi-membrane arrangement to make a spiral RO membrane used in this study is shown in Figure 3.

### 2.3. Membrane Samples: Cleaning and Storage

Fouled membrane pieces were cut 20 cm away from the edges of Rack J P11-1 and Rack J P11-2 (named as P1 and P2) membrane elements. P1 and P2 membrane pieces were preserved in deionized water (DI) for 30 min before being treated with acid and base solutions, and before bacteria isolation (see below). To avoid the cross-contamination of bacteria from the ambient, prepared membranes were quickly transferred to the biological lab. In order to identify cleanable fouling, some coupons from P1 and P2 were dipped in 6% *w*/*v* citric acid and 6% *w*/*v* sodium hydroxide for 30 min, followed by dipping in DI water for 30 min. All membranes were dried at room temperature and kept in an electronic desiccator for two days before characterization.

### 2.4. Membrane Samples Characterization

The fouled, cleaned, and virgin membranes visual inspection was conducted using optical images taken with a commercial camera in reflection mode. The surface morphology of all membranes was examined using a field-emission scanning electron microscope (FESEM, JEOL JSM-7600F) operated at an accelerated voltage of 15 kV with working distance of 10 mm. Energy-dispersive X-ray spectroscopy (EDXS) with AZtec 3.0 software detectors (EDXS, Oxford instrument X-Max, UK) was used for elemental mapping of the membranes. Data were interpreted using AZtec nanoanalysis software. The crystalline nature of all membranes was characterized using X-ray diffraction (XRD, Miniflex 600, Rigaku, Tokyo, Japan) with Cu Ka radiation in the scanning range from 10° to 90° in 0.05°/s steps. The surface functional groups of all membranes were studied by attenuated total reflection–Fourier transforms infrared (ATR-FTIR) spectroscopy (PerkinElmer, Spectra One, Waltham, MA, USA). The spectra were recorded at a resolution of 4.0 cm^−1^ in the frequency region of 4000–500 cm^−1^, with an average of 40 scans per sample. The organic and inorganic contents in membranes were investigated by thermal gravimetric analysis (TGA) using Perkin Elmer (STA 6000, Waltham, MA, USA) analyzer under N_2_ gas at a heating rate of 10 °C/min and a gas flow of 20 mL/min from 25 °C to 900 °C. Surface chemistry of the virgin, fouled, and cleaned membrane surfaces was investigated using an X-ray photoelectron spectroscopy (XPS) technique (Scienta Omicron, Taunesstein, Germany). Since the membrane material was an insulator, a stream of electrons was bombarded onto the sample surface during XPS measurements to compensate for the charging effect. Quantitative and qualitative analyses of the XPS results were carried out using Casa XPS software (Casa Software Ltd., Terrace, Teignmouth, UK). Intrinsic carbon with a binding energy value of 284.6 eV was used as reference binding energy and calibrated accordingly.

### 2.5. Bacteria Isolation and Identification

The isolation and identification of bacteria were performed under sterile conditions in the laboratory. First, Marine Broth 2216 (Merck, Darmstadt, Germany) was prepared according to the manual. Then, 9 mL of marine broth was placed in each culture tube and autoclaved (temperature = 122 °C, time = 15 min). Second, three pieces (1 × 1 cm) from randomly selected places of each membrane were cut using sterile scissors. Each piece was placed individually in a culture tube. Third, the tubes were incubated at 37 °C for 48 h. After that, 1 mL of spent broth was transferred onto a Zobell marine agar (HiMedia, West Chester, PA, USA) and incubated for a further 24 h at 37 °C. Fifth, in order to isolate pure bacterial strains, individual colonies were collected using sterilized metal loop and streaked on a new marine agar Petri dish. These dishes were incubated at 37 °C for 24 h. This process was repeated several times until pure colonies of bacteria were obtained. Finally, pure bacteria colonies were identified using MALDI-TOF (matrix-assisted laser desorption ionization/time of flight) on a Microflex^®^ LRF TOF/TOF mass spectrometer (Bruker Daltonics, Leipzig, Germany). This method determined the unique proteomic fingerprint of a bacterium. The mass spectrometer was calibrated using the Bruker bacterial test standard (*Escherichia coli*) and run following the manufacturer’s instructions. The mass spectral data obtained from each bacterial isolate were analyzed using the BioTyper software (Bruker, Bremen, Germany) to identify bacteria. All samples were analyzed in duplicates. In the case of contradictory results, the identification of bacteria was repeated until a clear identity was obtained. 

## 3. Results and Discussion

### 3.1. Optical Analysis of Fouled and Cleaned Membranes

**Figure 4:**
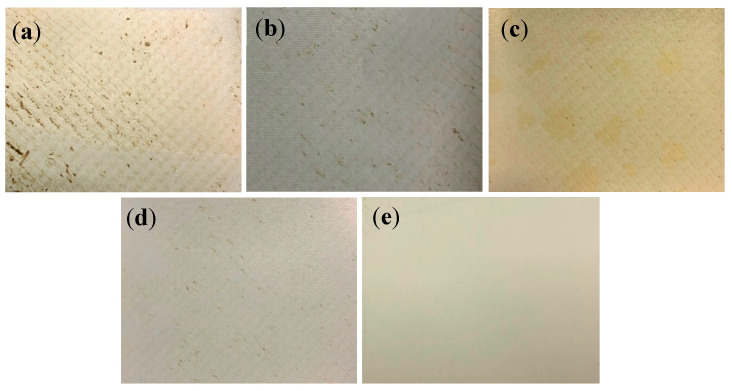
Optical images of fouled and cleaned membranes of (**a**) P1, (**b**) P2, (**c**) P1-clean, (**d**) P2-clean, and (**e**) the virgin membrane.

Visual inspection is one of the most import aspects to study the physical surface nature of the fouled membrane. This visual inspection also helps to understand the nature of fouling. For instance, we can note the presence of foulants by observing the color of the membrane surface. Thus, prior to the investigation with FESEM, optical images were taken, and visual inspections were carried out. Figure 4 shows the optical images of the fouled (P1 and P2), cleaned (P1-clean and P2-clean), and virgin membranes. The surfaces of the fouled P1 and P2 membranes were randomly covered with brown-colored deposits (see Figure 4a,b) that were not heavily fouled. After cleaning with acid and base media (immersed in the acid and base media at room temperature without any agitation or mechanical scrubbing), most of the deposits were removed, as shown in the figure for P1-clean and P2-clean (Figure 4c,d). However, some of the scale precipitates were not entirely removed, and the brown precipitates still existed on the membrane surface, as shown in Figure 4c,d. These leftover precipitates were further investigated with FESEM and EDXS methods. The virgin membrane in Figure 4e shows a clean and smooth surface in nature.

### 3.2. FESEM Analysis of Fouled and Cleaned Membranes

**Figure 5:**
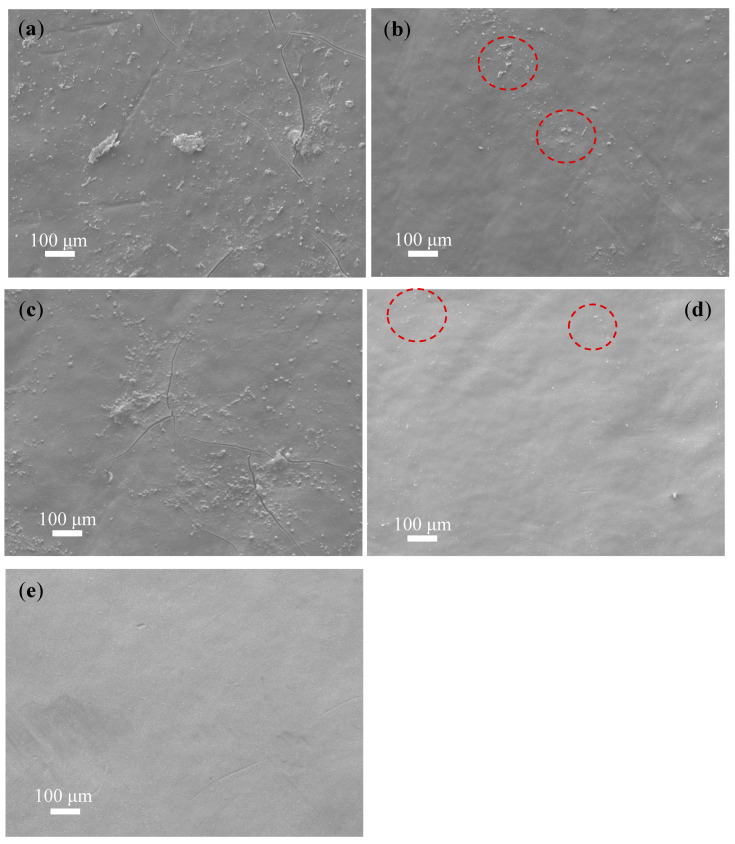
SEM images of fouled and cleaned membranes of (**a**) P1, (**b**) P1-clean, (**c**) P2, (**d**) P2-clean, and (**e**) virgin membrane.

Figure 5a–d show FESEM images of fouled membranes before and after cleaning with acid and base media, while Figure 4e shows the virgin membrane for comparison. The surface morphology of the fouled and virgin membranes were quite different, where the fouled membranes (P1 and P2) had rougher surfaces with crevices and the virgin membrane had a smooth and clean surface. The fouled P1 and P2 membrane surfaces (Figure 5a,c) were randomly covered with different shapes and sizes of precipitates, which could have been due to the inorganic foulants known as scaling [35]. These foulants were later confirmed with elemental analysis using EDXS. Moreover, the cracks in some parts of the fouled membranes, Figure 5a–c, may have occurred because of the deposition of different foulant materials on the membrane surface. These cracks were not visually observed in optical images due to the smaller size of the cracks (~100 to 200 µm in length). In order to investigate the elemental composition of the foulants present on the membrane surface, EDXS analysis was carried out on the virgin, fouled, and cleaned membranes. Since the EDXS technique is sub-surface (1–3 µm in-depth), the detected elements emerged from the polyamide and polysulfone layers (please see Figure 3b for the reference). For the fouled membranes (P1 and P2), various elements, such as C, O, S, Cl, Na, Al, Mg, and Ca, were detected (Figure S1 in Supplementary Material). These elements can be related to the deposition of inorganic salts such as NaCl and CaCO_3_, which are the major components of the feed water. After cleaning with acid and base media (see Figure 5b,d), fewer deposits were witnessed when compared with the uncleaned P1 and P2 membranes. For comparison, the virgin membrane was also investigated; its surface mainly consisted of C, O, S, and Al (Figure S3), and similar elements were also detected (Figure S2) on the P1-clean and P2- clean surfaces. However, some deposits (indicated by red circles in Figure 5b,d) were still present on the P1-clean (Figure 5b) and P2-clean (Figure 5d) membranes, even after cleaning with acid and base media. Moreover, it can be observed that the P1-clean membrane had more deposition than the P2-clean sample after the cleaning process. The presence of these deposits might have been due to the incomplete removal of strong bonding minerals on the membrane surface during the cleaning process or the re-deposition of residues from the cleaning solution. The differences in deposition were further confirmed using a deposit weight density test, as shown in Table S1. An EDXS analysis was further conducted on these deposits (indicated by red circles), and different elements including C, O, S, Si, Ca, Al, and Mg were detected (Figures S4 and S5), which can come from SiO_2_ and CaMg(CO_3_)_2_ [36].

### 3.3. XRD Analysis of Fouled and Cleaned Membranes

**Figure 6:**
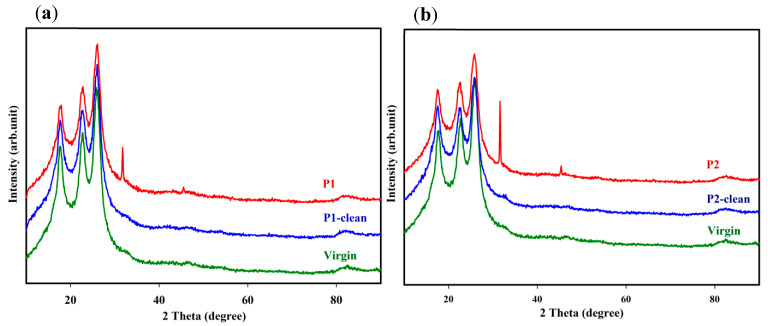
XRD diffraction patterns of fouled membranes (P1 and P2) and virgin membranes (**a**) before and (**b**) after cleaning with acid and base media.

An XRD analysis was conducted to study the crystalline and amorphous nature of the deposits on the fouled and cleaned membranes’ surfaces. Figure 6a,b show XRD patterns for the fouled membranes (P1 and P2), fouled membranes after cleaning (P1-clean and P2-clean), and virgin seawater membranes. It was found that three broad diffraction peaks appeared at 2θ = 17.5°, 22.5°, and 25.8° for all membranes obtained from the semicrystalline nature of the polyamide membrane [37]. For the fouled P1 and P2 membranes, two additional peaks were observed at 2θ = 31.5° and 45.5° due to the drying of NaCl from seawater as halite forms on the membrane surface [38,39]. The presence of NaCl on the fouled P1 and P2 membranes was also observed in the EDXS analysis and further confirmed using a quantitative analysis with XPS as shown in Section 3.6 Other elemental compounds observed in the EDXS analysis were not detected in the XRD analysis apart from the NaCl crystal, presumably due to their low concentrations on the membrane surface. After cleaning with the acid and base treatment, the crystalline phase deposits disappeared, and the XRD patterns for P1-clean and P2-clean were almost similar to those of the virgin membrane (Figure 6a,b).

### 3.4. FTIR Analysis of Fouled and Cleaned Membranes

**Figure 7:**
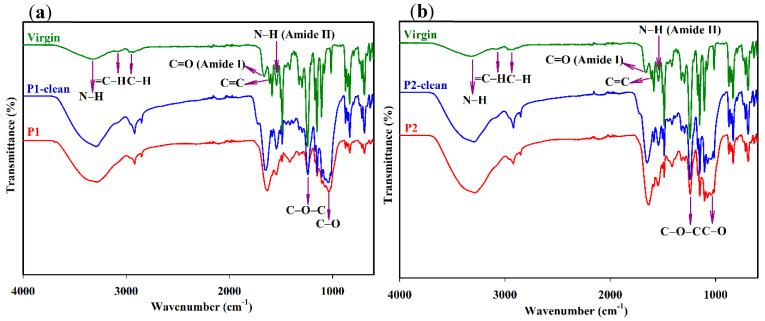
The FTIR spectra (**a**,**b**) of fouled membranes (P1 and P2) before and after cleaning with acid and base media and virgin membranes.

FTIR spectroscopy was used to investigate the organic compounds on the fouled membrane surfaces before and after the cleaning process. Different types of functional groups were detected from both the polyamide (surface layer) and the polysulfone layers (ATR-FTIR: effective depth of ~1 µm) at the lower wavenumber range between 800 and 1800 cm^−1^. At a higher wavenumber range between 2700 and 3700 cm^−1^, only the chemical characteristics of the surface layer (200 nm from the surface) can be identified [40]. Figure 7a,b show FTIR spectra for the fouled P1 and P2, P1-clean, P2-clean, and virgin membranes. Apparent changes in the wavenumber range of 800–1800 cm^−1^ appeared in both the P1 and P2 membranes compared to the virgin membrane. The individual peak at 1664 cm^−1^ was noted as an amide I band (C=O), and a C=C ring vibration of the polyamide layer at 1609 cm^−1^ [41] appeared as a single band around 1650–1630 cm^−1^ for both fouled P1 and P2 membranes, presumably due to protein fouling. In addition, a reduction in amide II band (N-H) intensity at 1544 cm^−1^ was observed for both P1 and P2 [41]. The changes in the band between 1484 cm^−1^ and 1410 cm^−1^ could have been due to aliphatic C-H deformation, C-O stretching, and the O-H deformation of phenol [24]. The band at 1243 cm^−1^ was a feature of the C-O-C asymmetric stretching vibration of the polysulfone support layer [40]. A new peak appearing at 1035 cm^−1^ for both fouled P1 and P2 membranes was attributed to a functional group of C-O obtained from the polysaccharides that caused fouling [42]. Figure 6a,b show the existence of this peak in the P1-clean and P2-clean membranes, even after cleaning with acid and base media. In addition, due to inorganic and aromatic fatty acid and protein fouling, a reduction in some bands at lower wavenumber regions (600–800 cm^−1^) was observed in the fouled membranes when compared to the P1-clean, P2-clean, and virgin membranes [24,43]. The bands between 2900 cm^−1^ and 3000 cm^−1^ were assigned to an aliphatic C-H stretching vibration, and the band around 3030–3090 cm^−1^ was attributed to an aromatic =C-H stretching vibration for all membranes [44]. The broad band at 3300 cm^−1^ was attributed to free and hydrogen bond N-H stretching vibrations. Moreover, the fouled P1 and P2 membranes showed a similar band at 3300 cm^−1^ with a more broad and increased intensity than that from the virgin membrane due to polysaccharide fouling [41], which contains numerous amounts of hydroxyl groups. The adhesive nature of polysaccharides could not be entirely removed by cleaning as an intense broad band at 2900–3300 cm^−1^ was detected for both P1-clean and P2-clean membranes. In addition, the polysaccharide material might behave as a trap for organic foulants and serve as a source of nutrients for bacterial growth on a membrane’s surface [45]. FTIR spectra from Figure 7a,b were compared with the literature, and the results are shown in Table 2.

### 3.5. TGA Analysis of Fouled and Cleaned Membranes

**Figure 8:**
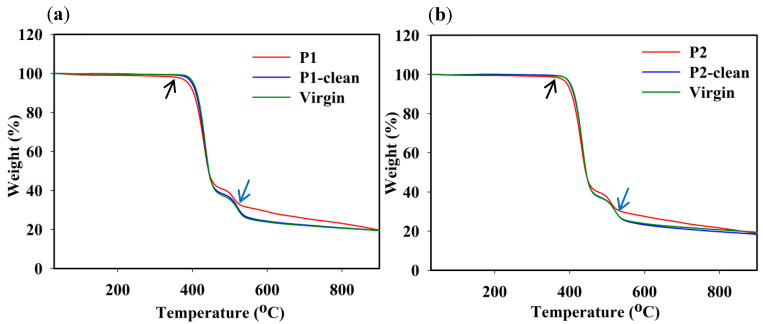
A comparison of TGA graphs for (**a**) P1 fouled, P1-clean, and virgin membrane and (**b**) P2 fouled, P2-clean, and virgin membrane.

In order to confirm the presence of different foulants such as organics and inorganics, TGA analysis carried out with the fouled membranes, cleaned membranes, and virgin membrane. Three weight-loss regions were observed for the fouled P1 and P2 membranes, whereas only two main weight-loss regions were observed for the P1-clean, P2-clean, and virgin membranes, as shown in Figure 8a,b. For the fouled membranes, the initial-stage weight loss of ~1.8% and ~1.2% for P1 and P2 appeared at 30–350 °C due to the adsorbed water and inorganic and organic foulants released from the membranes. However, the weight loss for the fouled P1 and P2 membranes around 350 °C appeared (indicated with a black arrow) faster than those for the P1-clean, P2-clean, and virgin membranes, indicating the presence of scale precipitates and various foulants, such as organic foulants (see FTIR analysis), on the surface. The second degradation for fouled P1 and P2, and the first degradation for the P1-clean, P2-clean, and virgin membranes, occurred at 350–450 °C. Fouled P1 and P2 lost ~46% and ~45 %, while P1-clean and P2-clean and the virgin membrane lost ~44%. This degradation was attributed to the membranes’ polymer degradation. The third degradation stage for fouled P1 and P2, and the second degradation stage for P1-clean, P2-clean, and the virgin membrane from 450 to 550 °C was due to the backbone cleavage of the polymer membranes [48]. In this stage, different foulants in uncleaned P1 and P2 resulted in an enhanced residue weight % (indicated with blue arrow), as shown in Figure 8a,b. However, the burn-off residues at 900 °C were ~20% for all membranes.

### 3.6. XPS Analysis of Fouled and Cleaned Membranes

**Figure 9:**
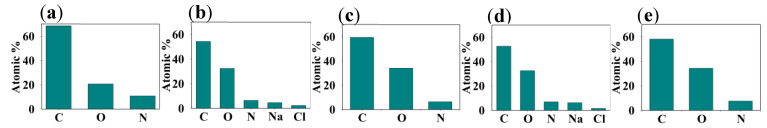
Quantitative analysis of elemental composition on the surface of (**a**) virgin, (**b**) P1, (**c**) P1-clean, (**d**) P2, and (**e**) P2-clean membranes.

In order to investigate the surface chemistry of the fouled, cleaned, and virgin membranes, the XPS technique was used, and a quantitative and qualitative analysis was performed. Figure 9a–e show the quantitative elemental analysis for virgin, fouled P1, P2, P1-clean, and P2-clean membranes estimated from a survey scan of the XPS analysis. XPS analysis is a surface-sensitive technique with a sampling depth of ~10 nm; thus, the elemental composition was different than the EDXS results. Generally, all of the membranes were predominantly composed of C, O, and N, which mainly arose from the polyamide layer (refer to Figure 3b). The virgin membrane was composed of 69% C, 20% O, and 11% N. The fouled P1 membrane surface consisted of 54% C, 32% O, 6% N, 5% Na, and 3% Cl (Figure 9b), while 53% C, 33% O, 6% N, 6% Na, and 2 % Cl were detected on the fouled P2 membrane surface (Figure 9d). The fouled P1 and P2 surfaces showed a substantial reduction in carbon content but higher surface oxygen content than the virgin membrane, which could have come from C=O formation on the membrane surface. This incremental scenario was in good agreement with the FTIR results, as shown in Figure 7a,b. Moreover, the virgin membrane had a higher C-O content than the fouled P1 and P2 membranes, which agreed with the FTIR results shown in Figure 6a,b as well. After cleaning with acid and base media, the C and N content slightly increased due to the removal of Na and Cl from the membrane surface (Figure 9c–e), whereby more photoelectron signals from the membrane active layer (polyamide) could be detected.

**Figure 10:**
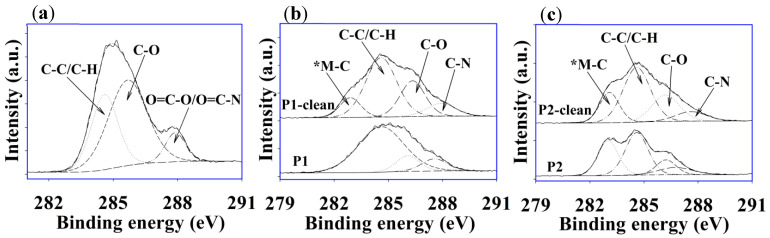
XPS high-resolution C 1s peak of (**a**) virgin, (**b**) P1 and P1-clean, and (**c**) P2 and P2-clean membranes.

Figure 10a–c show the high-resolution C 1s peak of the virgin, P1, P2, P1-clean, and P2-clean membranes acquired from the XPS measurement. The common component of C-C/C-H was observed at a BE value of 284.6 eV on all sample surfaces. However, a higher C-H content was observed for the fouled membrane compared with the virgin membrane, which could have been due to organic fouling. The FTIR and TGA results also supported this observation (see Figure 7a,b and Figure 8a,b). For the virgin membrane, carbon components of C-O and O=C-O or O=C-N were observed at BE values of 285.7 eV and 287.9 eV, respectively (Figure 10a) [49]. In Figure 10b, the fouled P1 membrane consisted of three C components, where C-C/C-H species dominated. Interestingly, four C components were detected on the fouled P2 membrane surface with an extra peak at the BE value of 283.1 eV (denoted as *M-C), which can be assigned as carbide-related impurities [50]. However, after cleaning the membrane surfaces (P1-clean and P2-clean), similar C components, such as C-C/C-H, C-O, and C=O/C-N, were witnessed. Surface C-O concentration increased after washing, which affirmed the removal of surface contamination/fouling and subsequent exposure of the original membrane surface (C-O content in the virgin membrane was the highest amongst all membranes).

### 3.7. Bacteria on Fouled and Cleaned Membranes

To investigate the biofouling on the membranes, bacterial isolation and identification were conducted. Table 3 shows the identity of bacterial isolates present on the P1, P1-clean, P2, and P2-clean membranes. No bacteria were isolated from the virgin membrane. Only Gram-positive bacteria belonging to the *Bacillus* genus were found on the fouled membranes. This supports the previous findings stating that *Bacillus* bacteria were commonly observed on RO membranes even after chlorine treatment [51]. This could have been due to the formation of spores by these bacteria. Five different strains were obtained from the fouled P1 and P2 membranes (see Table 3). The fouled P1 membrane had *Bacillus cereus*, while the fouled P2 membrane had *Bacillus cereus, Bacillus flexus*, and *Bacillus marisflavi* species. Most of the strains were isolated from the fouled membranes. The P1-clean membrane did not show the presence of bacteria. However, *Bacillus cereus* was found on the P2 membrane after cleaning (Table 3), presumably due to cross-contamination during the experimental process. Previous reports revealed that *Bacillus subtilis* and *Bacillus aquimaris* were mostly isolated from RO membranes [15,51,52]. The difference between our study could be explained by the difference in environmental and cleaning conditions. However, since no other genera of bacteria were isolated, our study highlighted the importance of the *Bacillus* species in membrane biofouling. 

## 4. Conclusions

The surfaces and sub-surfaces of virgin and fouled SWRO membranes from the Barka desalination plant were investigated to understand the fouling components and surface nature of RO membranes. The membrane autopsy revealed that inorganic scalants, such as Na, Cl, Mg, Al, and Ca, were present, although bacteria and organic matter were also detected. An XRD analysis of the fouled membranes before cleaning showed the semicrystalline nature of polyamides and the presence of deposited salt crystals. The presence of organic and inorganic foulants on the fouled and cleaned membranes was revealed by using FTIR for the surface and sub-surface, TGA for the whole membrane, and XPS. Bacteria identification results revealed different *Bacillus bacteria species, especially on the P2 fouled membrane*. From the results, it can be concluded that the fouling occurred due to either inorganic material, organic material, microbial foulant, or chemical oxidation that affected the membrane structure and physical properties. Therefore, optimized dosages of antiscalant and coagulant could be the best option for preventing the SWRO membrane fouling process. Moreover, the cleaning process did not completely remove the foulants from the membrane surface due to the presence of different types of fouling. This could be overcome by using destructive autopsy methods.

## Figures and Tables

**Table 1:** Seawater and product characteristics of Barka desalination plant.

Parameters	Concentration (ppm) Raw Water	Product Water
TDS (mg/L) pH Cond (µS/cm)	39,000–42,000 8.3 ± 0.2 55,714 to 60,000	<500 mg/L 8.15 to 8.2 891
Cations Potassium (K) Sodium (Na) Magnesium (Mg) Calcium (Ca)	408 mg/L 11,100 mg/L 1340 mg/L 435 mg/L	<0.1 mg/L 138 mg/L 2.3 mg/L 15 mg/L
Anions SO_4_^−2^ F^−^ Chloride as Cl^−^ HCO^−3^	3340 mg/L 2.74 mg/L 22,200 mg/L 168 mg/L	<5.0 mg/L <0.8 & >0.60 mg/L <250 mg/L
Boron	5.26	<2.4 mg/L

**Table 2:** Peak assignments for FTIR spectra from the literature.

Peaks	Peak Assignments and Features	References
1040 cm^−1^	C-O stretching, polysaccharides	Rahman et al. [42], Ashfaq et al. [43]
1243 cm^−1^	C-O-C, asymmetric stretching, polysulfone support layer	Shafi et al. [40]
1544 cm^−1^	N-H stretching, amide II band	Melián-Martel et al. [41], Matin et al. [46]
1609 cm^−1^	C=C ring vibration, polyamide layer	Melián-Martel et al. [41], Matin et al. [46]
1664 cm^−1^	C=O, amide I band, protein	Melián-Martel et al. [41], Matin et al. [46]
1484–1410 cm^−1^	aliphatic C-H deformation, C-O stretching, and O-H deformation of phenol	Tran et al. [24]
2900–3000 cm^−1^	aliphatic C-H stretching vibration	Lee et al. [44]
3030–3090 cm^−1^	aromatic =C-H stretching vibration	Lee et al. [44]
3300 cm^−1^	free and hydrogen bond N-H stretching, O-H stretching vibration	Melián-Martel et al. [41], Idarraga-Mora et al. [47]
600–800 cm^−1^	fatty acid, protein	Ashfaq et al. [43]
2900–3300 cm^−1^	polysaccharides	Ashfaq et al. [43]

**Table 3:** Bacterial isolates from fouled and cleaned membranes identified using MALDI-BIOTYPER analysis. The score provided by MALDI-Biotyper analysis: 0–1.69, unreliable identification; 1.70–1.99, probable genes identification; 2.0–2.29, secure genes identification and probable species identification; 2.3–3.0, confident species identification.

Sample	Strain Code	Bacterial Species	Mean Score Value
P1 fouled	P1 11	Unidentified	1.40
P1 fouled	P1 12	*Bacillus cereus*	2.12
P1 fouled	P1 2-2	*Bacillus cereus*	2.11
P1 fouled	P1 3-1	*Bacillus cereus*	1.87
P1 fouled	P1 3-2	*Bacillus cereus*	2.12
P2 fouled	P2 11	*Bacillus flexus*	1.73
P2 fouled	P2 12	*Bacillus cereus*	2.14
P2 fouled	P2 21	*Bacillus marisflavi*	1.73
P2 fouled	P2 22	*Bacillus cereus*	1.67
P2 fouled	P2 2-4	*Bacillus marisflavi*	1.72
P1-clean	No isolates	No isolates	
P2-clean	P2 Cl-2	*Bacillus cereus*	2.02

## Data Availability

The authors confirm that the data supporting the findings of this study are available.

## Supplementary Materials

The following supporting information can be downloaded at: https://www.mdpi.com/article/10.3390/membranes12070671/s1, Figure S1: SEM-EDX analysis of (a) P1 and (b) P2 membranes; Figure S2: SEM-EDX analysis of (a) P1-clean and (b) P2-clean membranes; Figure S3: SEM-EDX analysis of virgin sea water membrane; Figure S4: SEM-EDX analysis of some deposits attached on the P1-clean surface (a) spectrum 8 and (b) spectrum 9; Figure S5; SEM-EDX analysis of some deposits attached on the P2-clean surface (a) spectrum 18 and (b) spectrum 19.
