# Supplementary material for: Autopsy of Used Reverse Osmosis Membranes from the Largest Seawater Desalination Plant in Oman
Source: Membranes (Basel). 2022 Jun 28;12(7):671. doi: 10.3390/membranes12070671 (PMC9322904; doi:10.3390/membranes12070671)
Supplement: Supplementary file 1 [file membranes-12-00671-s001.zip › membranes-1770175-supplementary.pdf]

Autopsy of used reverse osmosis membranes from the largest seawater desalination plant in Oman

Mohammed Al-Abri[a,b*], Htet Htet Kyaw[a], Buthayna Al-Ghafri[a], Myo Tay Zar Myint[c], Sergey Dobretsov[d,e]

[a] Nanotechnology Research Center, Sultan Qaboos University, P.O. Box 33, Al-Khoud, Muscat 123, Oman

[b] Department of Petroleum and Chemical Engineering, College of Engineering, Sultan Qaboos University, P.O. Box 33, Al Khould, Muscat 123, Oman

[c] Department of Physics, College of Science, Sultan Qaboos University, P.O Box 36, Al-Khoud, Muscat 123, Oman

[d] Department of Marine Science and Fisheries, College of Agricultural & Marine Sciences, Sultan Qaboos University, PO Box 34, Al-Khoudh, Muscat 123, Oman

[e] Center of Excellence in Marine Biotechnology, Sultan Qaboos University, PO Box 50, Al-Khoudh, Muscat 123, Oman

*Corresponding Author: alabri@squ.edu.om

(a)

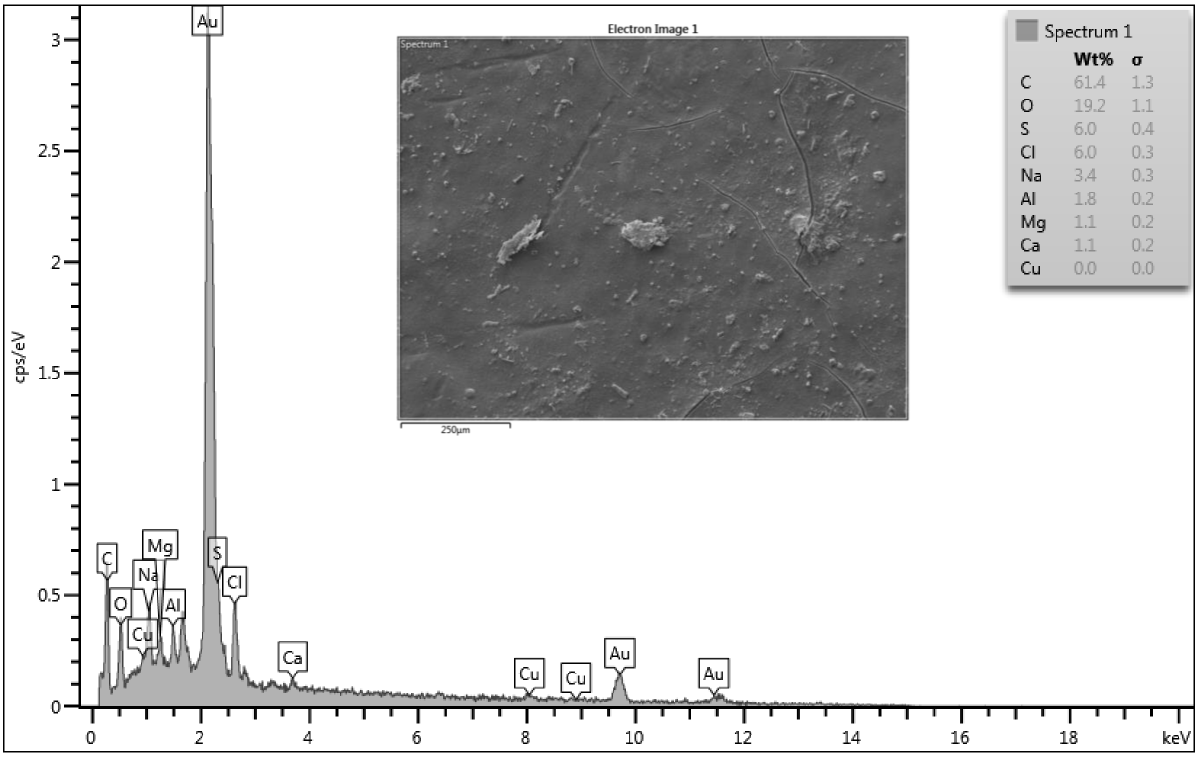

(b)

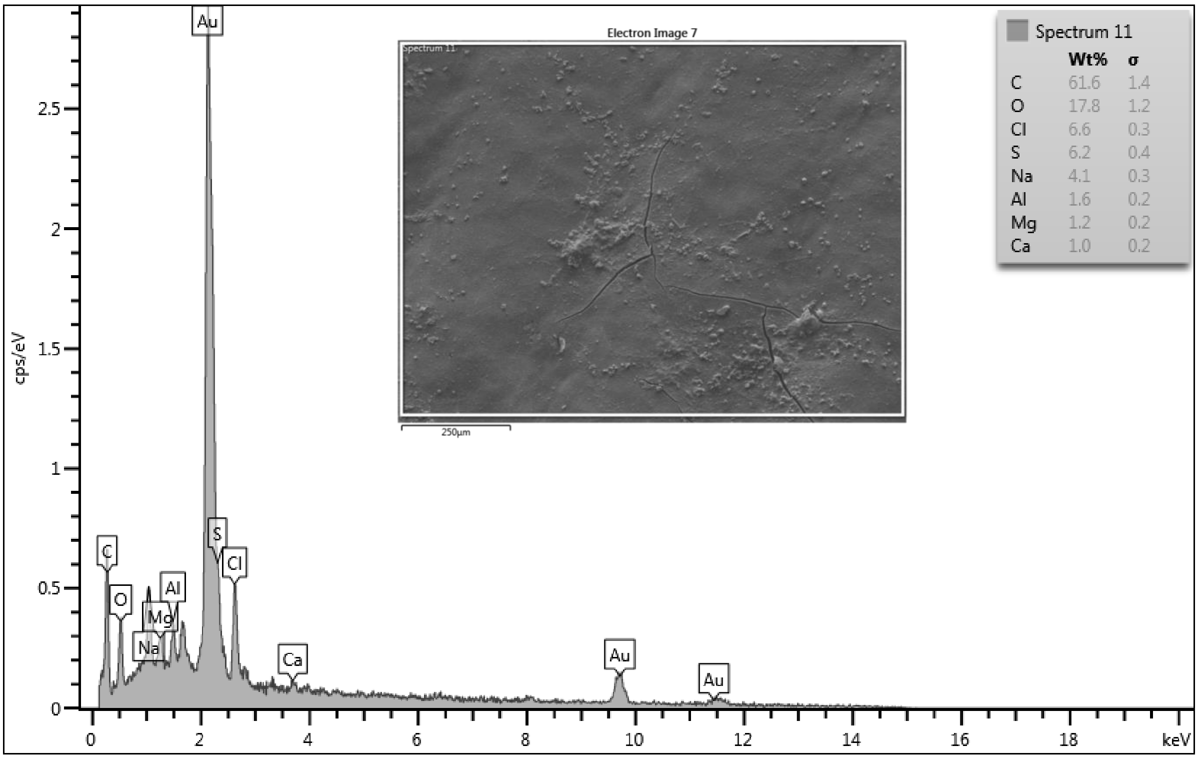

Figure S1. SEM-EDX analysis of (a) P1 and (b) P2 membranes.

(a)

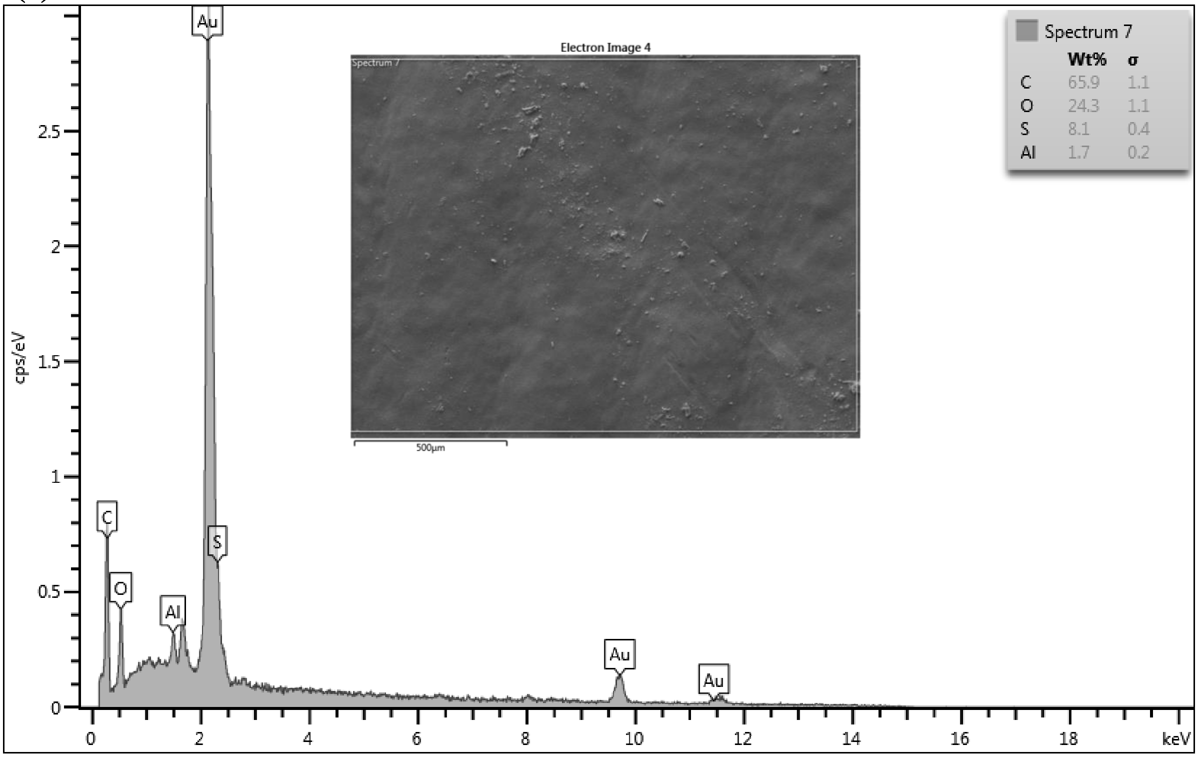

(b)

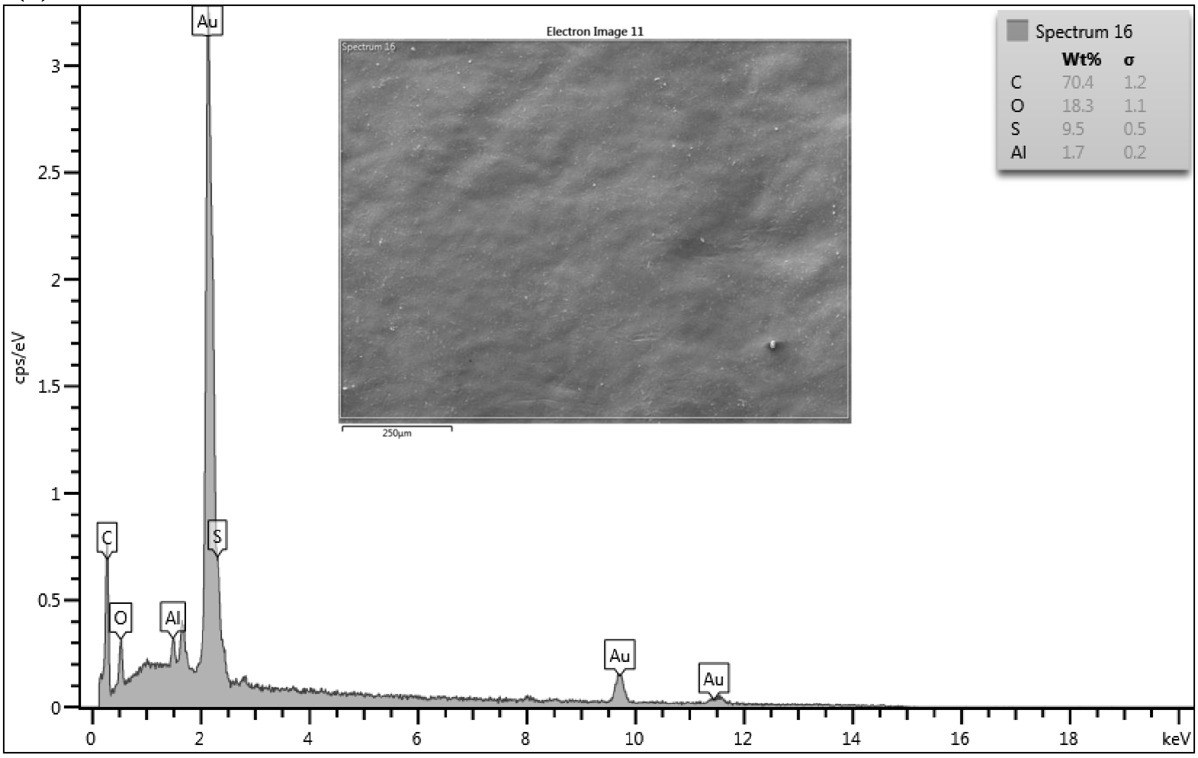

Figure S2. SEM-EDX analysis of (a) P1-clean and (b) P2-clean membranes.

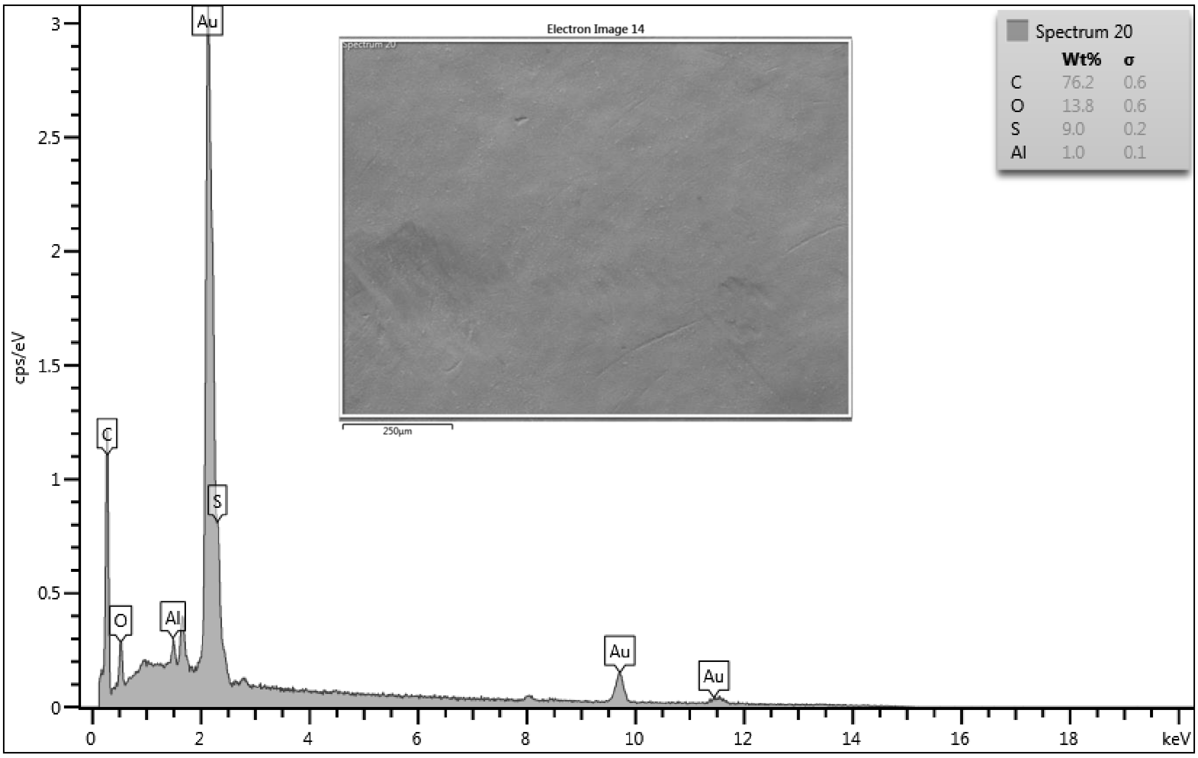

Figure S3. SEM-EDX analysis of virgin sea water membrane

(a)

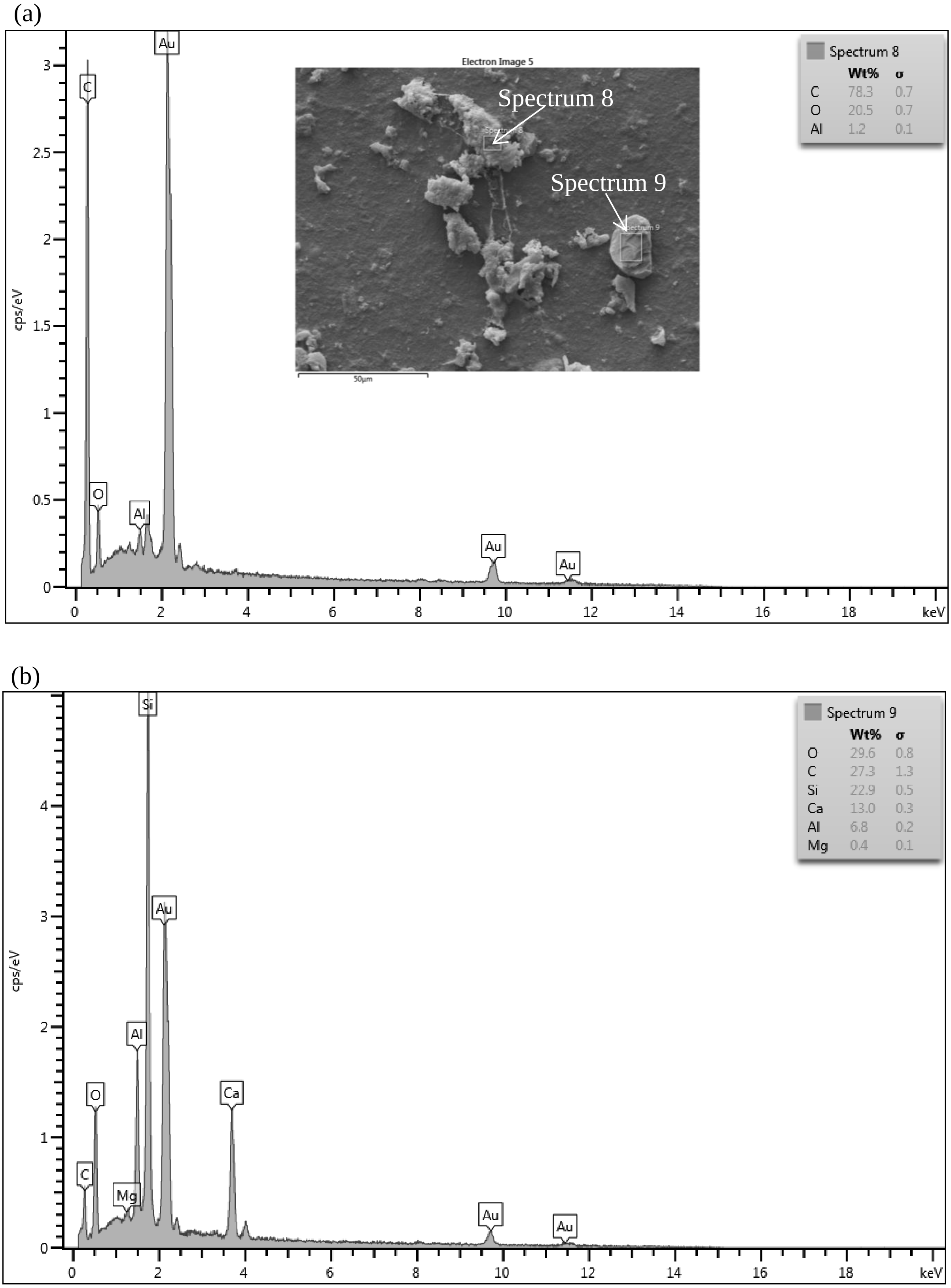

Figure S4. SEM-EDX analysis of some deposits attached on the P1-clean surface (a) spectrum 8 and (b) spectrum 9

(a)

(b)

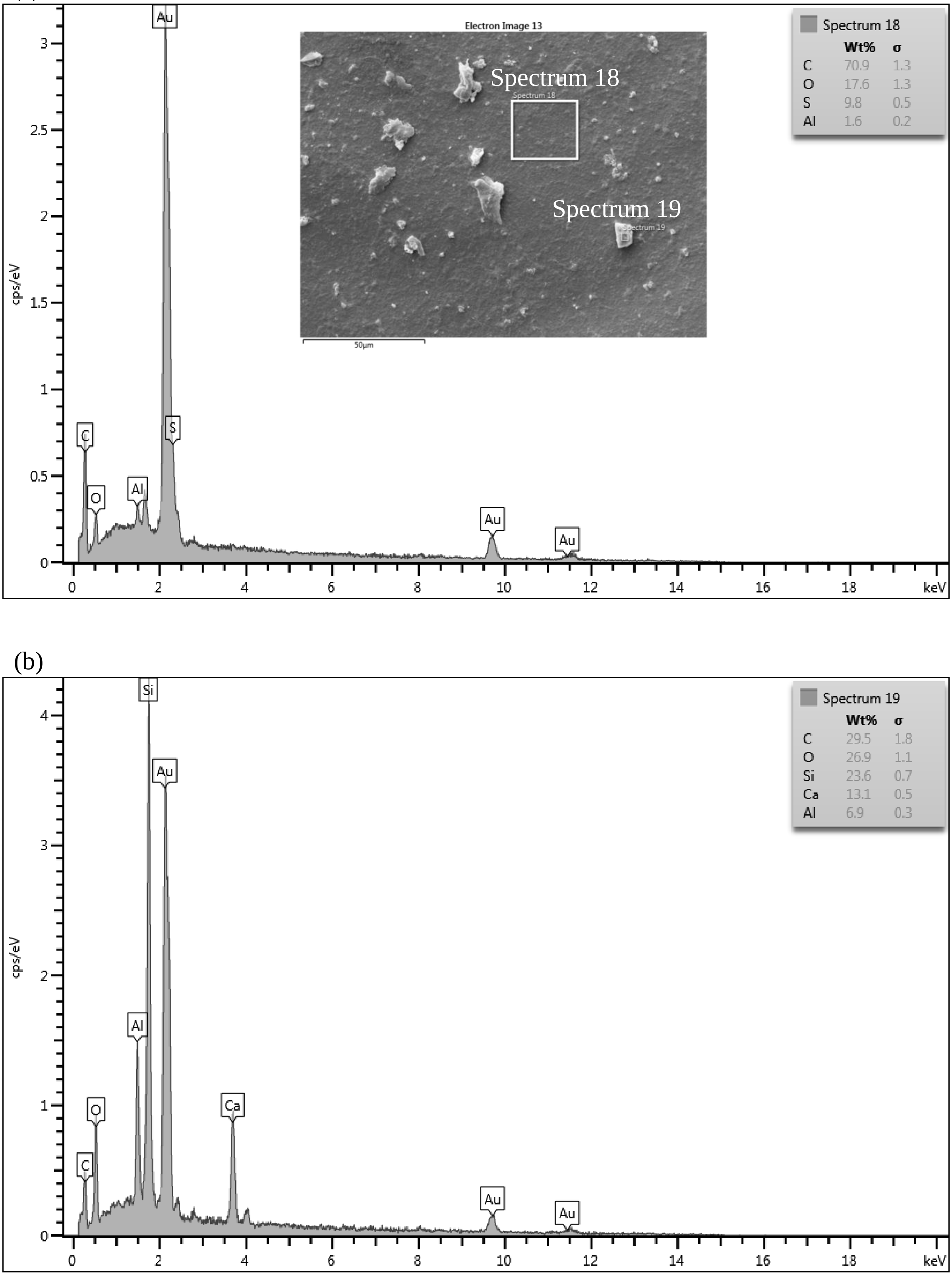

Figure S5. SEM-EDX analysis of some deposits attached on the P2-clean surface (a) spectrum 18 and (b) spectrum 19
